# Role of Physico-Chemical and Cellular Conditions on the Bone Repair Potential of Plastically Compressed Collagen Hydrogels

**DOI:** 10.3390/gels10020130

**Published:** 2024-02-06

**Authors:** Daline Mbitta Akoa, Ludovic Sicard, Christophe Hélary, Coralie Torrens, Brigitte Baroukh, Anne Poliard, Thibaud Coradin

**Affiliations:** 1 Sorbonne Université, CNRS, Laboratoire de Chimie de la Matière Condensée de Paris, 75005 Paris, France; daline.mbitta_akoa@sorbonne-universite.fr (D.M.A.); christophe.helary@sorbonne-universite.fr (C.H.); 2 Université de Paris, UR2496 Pathologies, Imagerie et Biothérapies Orofaciales, FHU-DDS-Net, Dental School, 92120 Montrouge, Francecoralie.torrens@parisdescartes.fr (C.T.); maya.baroukh@parisdescartes.fr (B.B.); anne.poliard@parisdescartes.fr (A.P.); 3 AP-HP Service de Médecine Bucco-Dentaire, Hôpital Bretonneau, 75018 Paris, France

**Keywords:** hydrogels, collagen, dental pulp stem cells, plastic compression, bone repair

## Abstract

Since their first description nearly 20 years ago, dense collagen hydrogels obtained by plastic compression have become popular scaffolds in tissue engineering. In particular, when seeded with dental pulp stem cells, they have demonstrated a great in vivo potential in cranial bone repair. Here, we investigated how physico-chemical and cell-seeding conditions could influence the formation and in vitro mineralization of these cellularized scaffolds. A qualitative assessment demonstrated that the gel stability before and after compression was highly sensitive to the conditions of fibrillogenesis, especially initial acid acetic and buffer concentrations. Gels with similar rheological properties but different fibrillar structures that exhibited different stabilities when used for the 3D culture of Stem cells from Human Exfoliated Deciduous teeth (SHEDs) could be prepared. Finally, in our optimal physico-chemical conditions, mineralization could be achieved only using human dental pulp stem cells (hDPSCs) at a high cell density. These results highlight the key role of fibrillogenic conditions and cell type/density on the bone repair potential of cell-laden plastically compressed collagen hydrogels.

## 1. Introduction

Type I collagen is one of the most popular biomolecules used to prepare materials for bone tissue engineering [1]. Although collagen materials can promote osteogenic differentiation, their bioactivity towards bone repair is most frequently improved following three main strategies: the formation of composite biomaterials by the incorporation of hydroxyapatite or bioglass particles; the loading of bioactive molecules, such as growth factors; or cellularization [2]. However, immobilizing cells in 3D environments whose density and organization somehow resemble the structure of living tissues is a major challenge [3,4]. Two main approaches can be followed: the colonization of pre-formed scaffolds or encapsulation, where the matrix is formed in the presence of cells [5]. The major advantage of colonization is that the scaffold can be prepared by a variety of processes that may not be cytocompatible, such as electrospinning [6]. However, achieving homogeneous cellularization of the scaffold can be challenging and time-consuming. In contrast, encapsulation allows cells to fill the scaffold from the very beginning of the culture period. However, this approach faces many challenges, including the cytocompatibility of the starting solutions, reagents, and processing conditions [7].

In the case of type I collagen, host matrices most often consist of hydrogels obtained by the neutralization of an acidic solution (pH 2–4) [8]. Therefore, in encapsulation approaches, the pH of the collagen solution must first be raised near neutral pH before cells suspended in an adequate buffer are included [9]. While this is feasible for collagen concentrations up to ca. 5 mg·mL^−1^ [10], higher concentrations lead to highly viscous, fast-gelling solutions, making adequate cell incorporation difficult [11]. More than 15 years ago, Brown and coll. disclosed an original protocol where cells were initially entrapped in a collagen hydrogel at a low concentration (ca. 2 mg·mL^−1^) followed by the densification of this hydrogel by at least a factor of 10 under plastic compression (PC) [12]. Therefore, this process allows for immobilizing cells in dense 3D scaffolds without requiring concentrated collagen solutions.

Initially, the effect of parameters such as loading force and time on the mechanical and morphological properties of the hydrogels, as well as their suitability to host human fibroblasts, were studied [12]. Further on, additional compression steps [13] and different geometries [14,15,16] were investigated. Finally, composite and mixed gels were described [17,18,19], and the range of immobilized cells was extended [20,21,22,23,24].

A feature of the PC process is its unique ability to create osteoid-like environments in a simple manner, which makes it an interesting alternative to additive manufacturing techniques [25]. It has, therefore, attracted much attention toward the design of bone repair materials, either by the chemical mineralization of the collagen hydrogels or by the immobilization of mineralizing cells [26]. In particular, PC hydrogels were shown to be suitable hosts for the encapsulation of stem cells derived from human dental pulp, promoting their osteo/odontogenic differentiation and leading to matrix mineralization in vitro [27]. Later on, it was demonstrated that such constructs could efficiently improve bone repair in vivo when implanted in critical defects of calvaria in rodents [28,29,30]. Noticeably, using fluorescent murine dental pulp stem cells (mDPSCs), it was shown that seeded cells could directly take part in neo-osteogenesis [31].

As this procedure became popular in the literature, several parameters, such as initial collagen solution content and neutralization routes, were modified [27,28]. The type (i.e., Stem cells from Human Exfoliated Deciduous teeth (SHEDs) or human dental pulp stem cells (hDPSCs) [32]) and density of the cells were also varied [27,28]. However, few studies investigated in detail the influence of such changes on the structural and biological properties of the gels [33,34]. In contrast, it was repeatedly demonstrated that type I collagen fibrillogenesis is highly dependent on pH, ionic strength, and salt type [8,35,36,37,38,39]. SHEDs and hDPSCs also demonstrated different mineralization capabilities in various environments [40,41,42]. On this basis, the present study aimed at understanding how physico-chemical and cellular conditions could impact the mineralization of PC dense hydrogels by DPSCs, a key requisite for their application as tissue engineering constructs for bone repair. Our results highlight that the range of physico-chemical conditions for which dense collagen hydrogels are able to sustain compression and cell-induced contraction is very narrow. Moreover, they demonstrate that the choice of pulp stem cell origin and density is a key factor in achieving mineralization in vitro.

## 2. Results and Discussion

### 2.1. Influence of Neutralization Conditions

**Figure 1:**
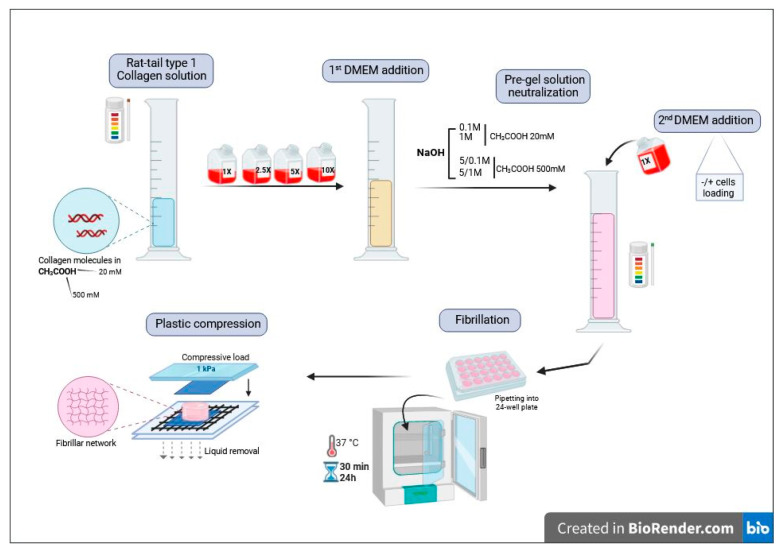
Schematic overview of the herein-studied multi-step preparation of plastically compressed collagen hydrogels.

In the first step, we qualitatively evaluated the effect of ionic strength and pH of the initial and neutralizing solutions on the structural stability of the hydrogels before and after PC (Figure 1). For this purpose, the concentration of acetic acid in the starting collagen I solution, the concentration and amount of the Dulbecco’s Modified Eagle Medium (DMEM) solution used before neutralization, and the concentration of the NaOH solutions used for neutralization were varied. The starting collagen solution volume was kept constant at 2 mL, and the final DMEM 1X addition was adjusted to reach a total volume of 5 mL. The loading stress and loading time were fixed to 1 kPa and 5 min, respectively, according to the literature [27].

**Figure 2:**
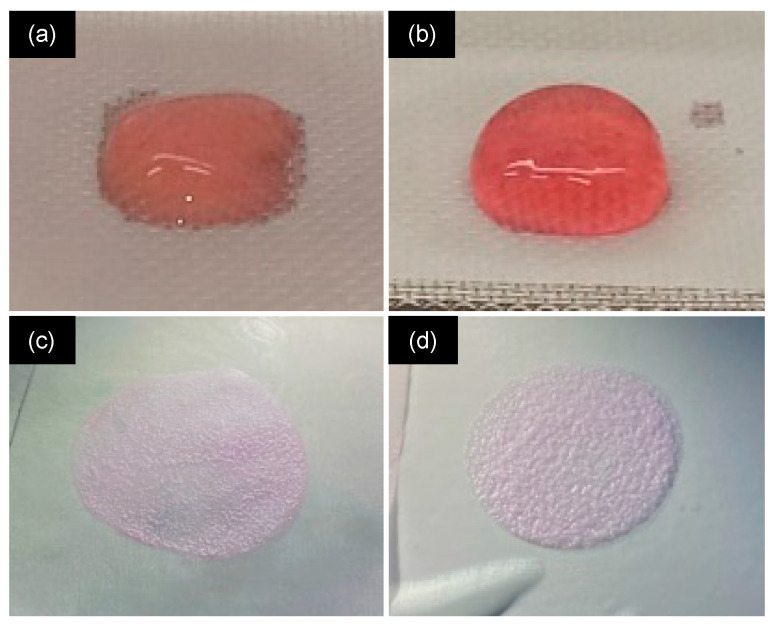
Macroscopic images of (**a**) a weak gel before compression; (**b**) stable gel before compression; (**c**) very thin gel after compression; and (**d**) thin gel after compression.

After 30 min of incubation at 37 °C, either no gel was formed, a weak gel that spread and leached was obtained (Figure 2a), or a stable gel was achieved that maintained its shape under its weight (Figure 2b). The gel either did not sustain the compression process or led to very thin (Figure 2c) or thin gels (Figure 2d) that were stable or broke when handled.

Starting from collagen in 20 mM acetic acid at pH 4 and using NaOH 0.1 M, adding 0.5 mL or 1.5 mL of DMEM at 1X or 2.5X led to weak gels before compression (Table 1). For a 0.5 mL addition, increasing DMEM concentration up to 10X led to stable gels before compression but resulted in very thin gels after PC. The addition of 1.5 mL of these concentrated DMEM solutions always yielded weak gels. In contrast, when 1 mL DMEM was added, the gel stability before and after compression increased when DMEM concentration increased from 1X to 5X. However, when 1 mL DMEM 10X was added, no gel was formed.

Starting from collagen in 500 mM acetic acid and keeping DMEM volume constant (1 mL), it was found to be necessary to perform the neutralization step via the successive addition of NaOH 5 M and NaOH 0.1 M (Table 2). In such conditions, only DMEM 2.5X yielded stable gels before and after compression. Again, in optimal conditions, increasing secondary NaOH concentration to 1 M also led to stable gels.

In summary, among all explored conditions, only three led to thin hydrogels that did not break after PC. Condition **H** involved low initial acetic acid concentration (20 mM), intermediate DMEM concentration (5X), and volume (1 mL) (Table 1). In condition **I**, a higher initial acetic acid concentration (500 mM) and a lower DMEM (2.5X) concentration were used, and both 5 M and 1 M NaOH solutions were required to reach neutral pH (Table 2). Note that the use of 0.1 M NaOH as a secondary neutralizing solution was also successful (second condition in Table 2). However, it implies the addition of a large volume of this solution and, therefore, a low volume of DMEM in the final step, which would require a highly concentrated cell suspension. This condition was, therefore, not further considered. Altogether, conditions **H** and **I** were the only ones producing hydrogels that could be easily handled without cracking and that were compatible with cell encapsulation.

### 2.2. Influence of Gel Aging Conditions

**Figure 3:**
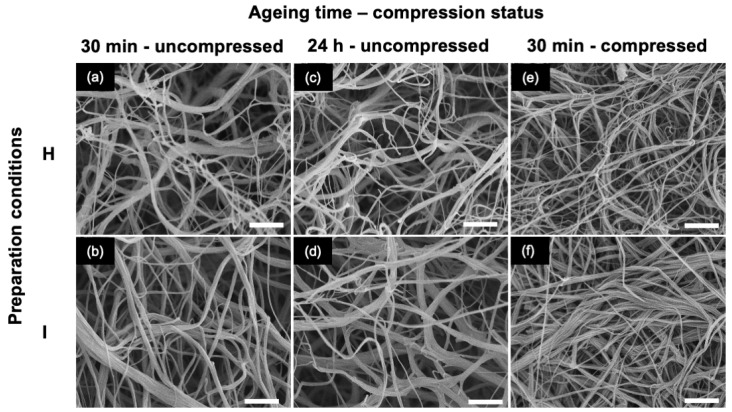
SEM images of hydrogels prepared in conditions H (top row **a**,**c**,**e**) and I (bottom row **b**,**d**,**f**), aged 30 min (left-hand column), aged 24 h (middle column) and aged 30 min and compressed (right-hand column). Scale bar: 2 μm.

In the second stage, the influence of gel aging before compression was studied for **H** and **I** conditions. Scanning electron microscopy (SEM) imaging of the hydrogels aged for 30 min showed that **H** consists of a porous network of highly intertwined thin fibrils, less than 100 nm in diameter, sometimes assembled as fibrous bundles up to 500 nm in diameter (Figure 3a). For sample **I**, a lower occurrence of thin fibrils could be observed, and the largest fibers could be up to 1 μm in diameter (Figure 3b). No clear modification could be observed for gels aged for 24 h, whatever the condition (Figure 3c,d). After compression, a top-view image of the gels aged for 30 min showed a decrease in the network porosity (Figure 3e,f). The largest bundles seemed partially unraveled, as particularly visible in condition I (Figure 3f). These observations were similar for gels aged for 30 min and 24 h (Figure S1).

**Figure 4:**
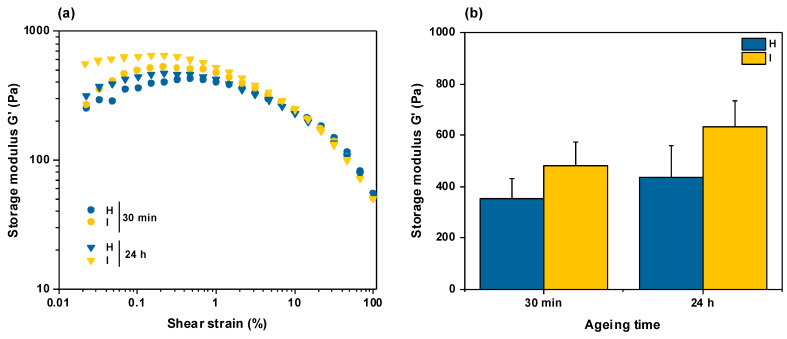
Rheological properties of hydrogels prepared in conditions H and I and compressed after 30 min or 24 h aging. (**a**) Evolution of storage modulus *G′* as a function of applied shear deformation at fixed frequency (10 Hz). (**b**) Influence of preparation condition and aging time on storage modulus *G′* at 0.1% shear deformation.

Compressed gels were also studied by rheology under shear stress. As seen in Figure 4a, gels compressed after 30 min or 24 h of aging exhibited similar behavior at a fixed frequency (10 Hz) and increasing strain, with an initial quasi-constant storage modulus *G′* value up to 1% deformation and then a continuous drop. When *G′* values at a 0.1% deformation were compared, the storage modulus was comparable for conditions **H** and **I** (Figure 4b). Noticeably, there was no significant difference between gels aged for 30 min and 24 h, so the former condition was selected for the rest of the work.

### 2.3. Influence of Cell Density

**Figure 5:**
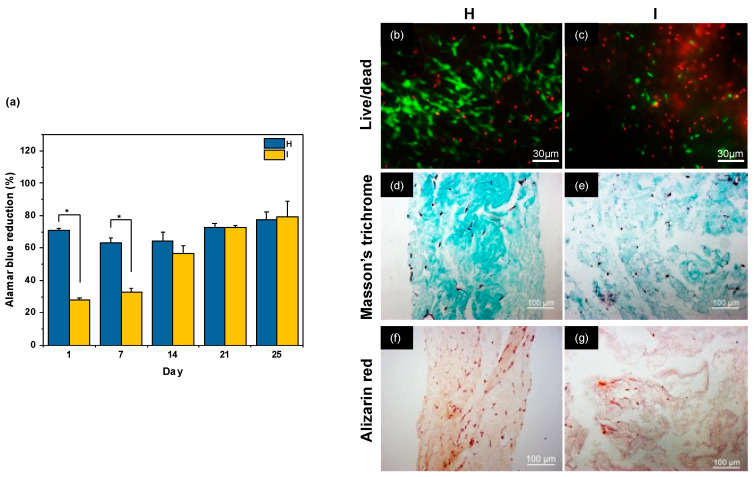
Metabolic activity, viability, and mineralization of SHEDs cells cultured within compressed collagen hydrogels prepared in conditions **H** and **I**. (**a**) Evolution of Alamar Blue reduction over 25 days of culture for the two conditions, (**b**,**c**) Live/Dead images of SHED cells after 25 days of culture. Green and red color are for live and dead cells, respectively. Scale bar: 30 µm. (**d**,**e**) Masson’s Trichrome and (**f**,**g**) Alizarin Red staining of SHED-cellularized hydrogels after 25 days of culture. * *p* < 0.05.

In the first experiment, SHEDs were encapsulated at a density of 160,000 cells·mL^−1^, and the gel was compressed after 30 min. After 25 days of culture, a significant shrinkage of both hydrogels was noticed, and upon recovery, the hydrogel obtained in condition **I** broke into fragments. A study of cell metabolic activity using the Alamar Blue test indicated that, in conditions **H**, cell activity remained constant over 25 days of culture (Figure 5a). In contrast, in condition **I**, the measured cell activity was small 1 day after compression and rise over the next three weeks to reach the value in **H**. Fluorescence imaging using the Live/Dead kit after 25 days in culture showed relatively few dead cells in gels obtained in condition **H** (Figure 5b) but a higher proportion of these were imaged in condition **I** (Figure 5c).

Masson’s Trichrome, which stains both collagen and cells, indicated that H consisted of a porous network with few scattered cells (Figure 5d). In condition I, large cavities were observed, which correlates with the observation of gel breaking into pieces (Figure 5e). Finally, staining with Alizarin Red (Figure 5f,g) and Von Kossa staining (Figure S2) showed no evidence of mineralization in any of the gels.

Based on this result, condition **H** was selected for the rest of the study as it offers an initial *G′* value comparable to **I** but formed a stable gel after 25 days of culture. Based on the literature [42], hDPSCs were used instead of SHEDs to favor mineralization. We also hypothesized that increasing cell density would increase their mineralization potential [43] so that higher densities of 800,000 cells·mL^−1^ and 2,000,000 cells·mL^−1^ were used.

**Figure 6:**
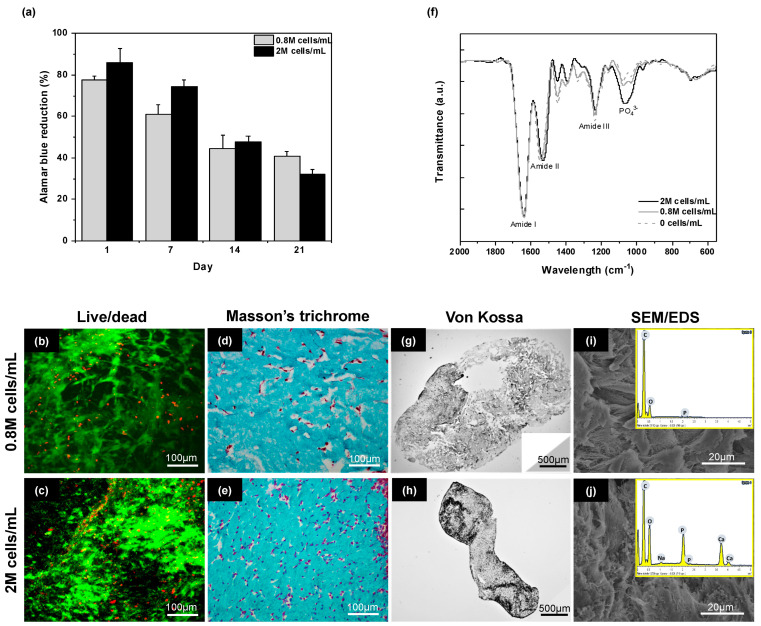
Metabolic activity, viability, and mineralization of hDPSCs cultured within compressed collagen hydrogels prepared in condition **H** with initial cell density of 0.8 M/mL and 2 M/mL under osteo/odontogenic conditions. (**a**) Evolution of Alamar Blue reduction over 25 days of culture for the two cell densities, (**b**,**c**) Live/Dead images of hDPSCs cells (green and red color are for alive and dead cells, respectively) and (**d**,**e**) Masson’s Trichrome staining of hydrogels sections after 25 days of culture. Scale bar: 100 µm. (**g**,**h**) Von Kossa staining, (**i**,**j**) SEM imaging and EDX analysis after 25 days of culture, (**f**) FTIR spectra of hydrogels after 25 days in culture conditions in the absence of cells and for 0.8 M·mL^−1^ or 2 M·mL^−1^ initial cell density.

For both initial cell densities, the Alamar Blue test showed a continuous decrease in cell activity over time (Figure 6a). After 25 days, many DPSCs cells could be observed with few dead cells (Figure 6b,c). For an initial 0.8 M·mL^−1^ content, Masson’s Trichrome staining revealed a dense gel with a higher cell density than in the previous conditions, as expected, but, except on the hydrogel edges, cells remained isolated (Figure 6d). In contrast, in the initial 2 M·mL^−1^ cell density condition, numerous cell–cell contacts could be evidenced within the dense collagen network (Figure 6e).

In terms of mineralization, for the lowest cell density, neither Von Kossa (Figure 6g) nor Alizarin Red (Figure S3) staining showed significant mineralization. No mineral deposit could be evidenced on SEM images, while Energy-Dispersive X-ray (EDX) spectroscopy did not allow for the detection of Calcium (Ca) or Phosphorus (P) (Figure 6i). In contrast, with the highest cell content, an important mineral deposit was observed in both stainings (Figure 6h and Figure S3). Such deposits could also be observed on SEM images, and EDX analyses confirmed that these particles contain Ca and P, with a Ca/P atomic ratio of ca. 1.5, thus close to the expected value for biological hydroxyapatite (Figure 6j). The presence of a well-defined vibration band at ca. 1100 cm^−1^ signing for the phosphate group in the hydroxyapatite structure on the Fourier Transform Infra-Red Spectroscopy (FTIR) spectrum of the latter sample only is consistent with our previous observations (Figure 6f).

### 2.4. Discussion

The aim of this study was to understand how chemical processing conditions and the origin/initial density of pulp stem cells could influence the mineralization of plastically compressed collagen hydrogels that demonstrated a strong potential for craniofacial bone repair [28]. In the first step, some key parameters of the PC process whose influence on final hydrogel structure and properties are yet to be studied in detail so far were selected and varied while keeping collagen initial and final concentrations and total volume constant.

In the pre-compression steps (Figure 1), the initial acetic acid concentration of the collagen solution not only dictates the pH and buffering capacity of the starting solution but also controls its initial ionic strength. The aim of the first DMEM addition is mainly to provide an adequate inorganic and organic composition favorable to cell survival. However, DMEM also influences the ionic strength and pH of the solution. Further addition of NaOH is required to obtain a precise pH value (7.4) common to all experiments. The last addition of DMEM allows for reaching a final sample volume identical for all conditions and, hence, a similar collagen concentration and also allows for the incorporation of the cell suspension when necessary. Therefore, the concentrations of acetic acid and DMEM are key factors determining the physico-chemical conditions of fibrillogenesis. In the case of the NaOH solution, its concentration determines the volume needed to achieve neutralization, which, in turn, dictates the volume of DMEM/cell suspension added at the last step, therefore being relevant for both physico-chemical and cellular conditions.

In the PC process, the collagen concentration before compression was fixed to 1.6 mg·mL^−1^, which corresponds to the conditions initially described to prepare cellularized soft hydrogels [12]. The starting collagen solution is usually 2–2.1 mg·mL^−1^, meaning that only small DMEM and NaOH volumes can be added. Here, a 4 mg·mL^−1^ starting concentration was selected so as to explore a large range of processing conditions. In terms of acetic acid, earlier reports used 20–100 mM concentration [12,28]. Here, to explore in more detail the role of ionic strength, a low- (20 mM) and a high- (500 mM) acetic acid concentration were selected.

Qualitative evaluation of the influence of initial acetic acid concentration, DMEM volume and concentration, and neutralization step on gel formation and compression showed that for 20 mM acetic acid, low-DMEM concentration or high-DMEM volume led to weak gels. These conditions correspond to the highest pHs before neutralization (Table 1). It was reported that increasing pH favors fibrillogenesis up to ca. pH 9, corresponding to the isoelectric point of type I collagen [38]. In acidic conditions, collagen triple helices are highly positively charged and cannot assemble due to electrostatic repulsion. As the pH increases, the positive charge decreases, and assembly can proceed. The smaller the charge, the faster triple helices assemble, which, according to the nucleation/growth model, results in the formation of numerous small fibrils [44]. The stability of the network is determined by inter-fibrillar interactions, and the contact area between two fibrils decreases with the fibril size [45]. Thus, it can be suggested that, in these situations where the pH is already high before the addition of NaOH, small collagen fibrils are rapidly formed, leading to loose networks. In contrast, all gels that were found stable before compression are formed for a pre-neutralization pH of 3 (Table 1 and Table 2), i.e., when collagen triple helices do not self-assemble [46]. It is important to note that the pH of DMEM solution decreases with increasing concentration, i.e., DMEM 1X has a pH of 8, whereas DMEM 10X has a pH of 2. This explains why the addition of DMEM before neutralization can lead to solutions that are more acidic than the initial collagen solution.

In addition, for similar pHs before neutralization, there seems to be an optimal initial DMEM concentration and volume. For instance, for 20 mM acetic acid, conditions (0.5 mL; 10X) and (1.0 mL; 5X) lead to similar pH (3), same required NaOH volume addition (700 μL), and close final DMEM concentration (1.3–1.4X), but only the latter yields to thin and stable gels after compression. Thus, the ionic content of these solutions before neutralization also impacts the fibrillogenesis occurring upon NaOH addition, in agreement with the literature [39]. This is because the presence of salts leads to the screening of the positive charges of collagen triple helices, favoring their assembly. When considering 500 mM acetic acid, an optimal initial DMEM concentration (2.5X) at fixed added volume (1 mL) was found. Importantly, this condition corresponds to a pH value of 3 before neutralization, emphasizing again the key importance of this value, and more globally, of the first addition step. It can be noticed that, although some of the conditions explored here would probably not be suitable for cell survival, the three optimal conditions lead to a final DMEM concentration close to 1X, i.e., the usual concentration for SHEDs and DPSCs culture [32].

More detailed investigations of samples **H** (prepared from 20 mM acetic acid) and **I** (prepared from 500 mM acetic acid) provide additional information about the impact of preparation conditions on gel properties. From a structural point of view, the difference between **H** and **I** in terms of fibrillar organization, i.e., thin fibrils with small bundles for the former compared to larger fibers for the latter, points to initial acetic acid concentration as a key parameter in the fibrillogenesis course. This is consistent with previous reports showing that high ionic strength favors collagen fibrils aggregation by decreasing their repulsive electrostatic interactions via charge screening [37,38,45]. It is important to note that the collagen concentration before the final DMEM addition was very close in the two conditions (2.4 mg·mL^−1^ for **H** compared to 2.2 mg·mL^−1^ for **I**), so the observed structural difference is unlikely to be related to this parameter. Interestingly, these larger fibers appeared significantly disturbed by the compression process, which confirms that they are weak physical aggregates. However, such a structural difference had little impact on the rheological properties of the hydrogels as there was no significant difference in *G′* values found for **H** and **I** samples. Finally, it can be emphasized that neither SEM nor rheology suggested any modification of interfibrillar interactions when increasing gel aging before compression. This indicates that a stable structure is already obtained after 30 min in the investigated conditions.

Stem cells isolated from the dental pulp attract much interest in the field of tissue engineering, especially because of their easy access [47]. The difference between SHEDs and hDPSCs originates from the maturity of pulp tissue they are isolated from, i.e., deciduous and permanent teeth, respectively [32]. As a consequence, SHEDs show a higher proliferation capacity, whereas DPSCs demonstrate a higher osteogenic/odontogenic differentiation potential [42]. Our initial conditions for mineralization experiments were based on the literature that used the initial PC protocol with SHEDs at a cell density of 150,000 cells per mL [27]. In this former work, cell activity was not studied, and cell viability was reported up to day 16 only, showing a limited number of dead cells via the Live/Dead staining kit. However, high levels of alkaline phosphatase and mineralizing proteins were detected, while the formation of hydroxyapatite was confirmed by FTIR. In our experiments, no significant mineralization could be observed by Von Kossa and Alizarin staining. In condition **H**, we observed that the cell activity was slightly increasing over time while very few dead cells were observed, indicating a slow proliferation of the cells. It is worth pointing out that the matrix average pore size, as estimated from SEM images of the acellular scaffold, is below 5 μm. This indicates that cells cannot simply migrate through the porous network but first need to degrade the matrix, explaining the low proliferation rate. In contrast, in condition **I**, the initial cell activity was very low, but with time, a large increase in cell activity was observed, indicating fast proliferation of the SHEDs. Considering that Live/Dead imaging showed a high proportion of dead cells within the gel and that the corresponding gel tends to break into pieces, it can be suggested that such high proliferation corresponds to unencapsulated cells proliferating on plastic walls of the wells, whereas very few survived inside the gels. In other words, in condition **H,** cells remain within the gel and slowly proliferate. The low ratio of dead/live cells suggests that they are undergoing a differentiation stage. In contrast, in condition **I,** the gel breaks, and some cells can move to the culture plate. In this situation, cells within the gel pieces die (high dead/live cell ratio), while those outside can freely proliferate (high metabolic activity).

The structural evolution of the gels over the culture period can be related to the cell-mediated remodeling activity, which involves contraction, degradation, and extracellular matrix deposition [48]. These processes are highly dependent on the hydrogel composition, structure, and mechanical properties, which can also impact stem cell differentiation. In particular, the mechanical properties of the hydrogel are usually considered a key factor [49]. However, **H** and **I** have similar *G′* values but evolved very differently: **H** became denser, as expected under contraction, whereas **I** broke into pieces. One possible explanation may be related to the difference in structural homogeneity of the starting gels (Figure 3). If the original network is homogenous, contraction will occur similarly within the whole gel, leading to a uniformly dense network, as observed in **H**. In contrast, if the network is initially heterogeneous, as in **I**, contraction may occur preferentially in some areas, creating zones of high and low density that can lead to local porosity and, ultimately, gel cracking.

On this basis, condition **H** was considered the most suitable, and in order to achieve mineralization, both cell type and cell density were modified. As pointed out above, hDPSCs were reported to have a lower proliferative but a higher osteogenic differentiation and, hence, mineralization potential compared to SHEDs [42]. The former point is confirmed by our cell activity measurement, showing a continuous decrease with culture time, whereas few dead cells could be observed by Live/Dead staining. However, a 0.8 M·mL^−1^ cell density did not allow the detection of mineral deposition. In contrast, when cell density was increased to 2 M·mL^−1^, histochemical staining, SEM/EDX, and FTIR evidenced mineralization within the gel. At the same time, such an increase in cell density also allowed for cell–cell contact in the gel, which is known to favor differentiation and mineralization [50]. The influence of cell density on mineralization within PC collagen hydrogels was already studied using a human osteosarcoma cell line (MG63) [51]. This work highlighted that cell density influences proliferation, differentiation, as well as hydrogel remodeling. Noticeably, the best cell density found here is comparable to the one used when DPSC-seeded PC collagen hydrogels were found to efficiently promote the repair of calvaria bone critical defects in rodents [28].

## 3. Conclusions

Here, we demonstrated that the properties of plastically compressed collagen hydrogels, including their suitability as cellular hosts, are highly sensitive to small modifications of the preparation protocol, especially in the acetic acid concentration of the collagen solution and the amount/concentration of cell culture medium. In addition, we highlighted that cell type and density were critical to achieving the mineralization of these dense collagen hydrogels by pulp stem cells in vitro. These results should provide useful guidelines for the further application of the plastic compression process in the fabrication of cell-laden tissue engineering constructs.

## 4. Materials and Methods

### 4.1. Materials and Cells

Type I collagen was extracted and purified from young rat tail tendon [37]. Young rat tails employed in this investigation were sourced as post-surgical discards from the animal facilities of UR2496 (Université Paris Cité), allowing for the utilization of otherwise discarded biological material in accordance with ethical and responsible laboratory practices. Dental pulps were obtained from exfoliated deciduous teeth of children aged 6–7 years old to isolate SHEDs or from human third molars of young adults (aged 15–20 years) to isolate hDPSCs. Teeth utilized in this study were obtained in the dental department of the AH-HP Bretonneau Hospital under an opt-out consent model, where patients undergoing surgical procedures were informed of the research use of excised tissues, and consent was presumed unless patients explicitly communicated their objection to such usage, in accordance with the ethical guidelines established by the French law on bioethics (agreement IRB 00006477 and n° DC-2009-927, Cellule Bioéthique DGRI/A5). The SHEDs and hDPSCs were isolated and expanded following an established protocol [28]. For all experiments, pulp cells were used in passage 4.

### 4.2. Plastically Compressed Collagen Hydrogel Preparation

In a typical experiment, 2 mL of a solution of type I collagen (4 mg·mL^−1^) in acetic acid (20 mM or 500 mM) was mixed with 0.5, 1.0, or 1.5 mL of DMEM of concentration varying between 1X and 10X in a falcon tube. Then, an aqueous solution of NaOH of concentration varying between 0.1 M and 5 M was added until pH 7.4, as monitored by a pH-meter. Finally, DMEM 1X was added so as to obtain a total 5 mL volume, and the solution was placed into 24-well plates (2.5 mL/well) and incubated at 37 °C under 5% CO_2_ atmosphere for 30 min or 24 h. Recovered collagen gels were deposited on a stack of blotting paper, nylon, and stainless-steel mesh and compressed/loaded with an unconfined compressive stress of 1 kPa using a calibrated glass plate for 5 min, as previously described [12,27] (Figure 1). 

### 4.3. Cell Culture Conditions

As previously described, after neutralization of the collagen mixture, two types of dental pulp stem cells at different cell densities were seeded in the collagen mixture prior to incubation: SHEDs: 160,000 cells·mL^−1^ [27], hDPSCs: 0.8 M cells·mL^−1^, and 2 M cells·mL^−1^ [28]. Gelling was followed by the plastic compression procedure described above.

For differentiation/mineralization studies, all cell-laden (with SHEDs or hDPSCs) plastically compressed dense collagen hydrogels were cultured in an osteo/odontogenic medium made of 300 µM L-ascorbic acid sodium salt, 10 nM dexamethasone, 10 mM β-glycerophosphate, 10% fetal bovine serum, and 1% penicillin/streptomycin for 25 days. Cell-free gels grown in this mineralizing medium were used as negative controls.

### 4.4. Structural and Chemical Characterizations

#### 4.4.1. Scanning Electron Microscopy (SEM)/Energy-Dispersive X-ray (EDX) Spectroscopy

Gels were fixed with 4% paraformaldehyde (PFA) in phosphate-buffered saline (PBS) and rinsed with a 0.1 M cacodylate/0.6 M sucrose buffer. They were dehydrated in aqueous solutions of increasing ethanol content and dried using supercritical CO_2_. Dried samples were sputter-coated with a 15 nm gold layer for SEM images and a 20 nm carbon layer for EDX analysis. SEM images were taken at several sites on each sample using a Hitachi S-3400 N microscope operating at 10 kV, while chemical characterization by EDX was performed at 60 kV.

#### 4.4.2. Rheological Studies

Rheological analysis of collagen hydrogels was carried out using a rheometer (Anton Paar) equipped with parallel, roughened 25 mm diameter stainless steel plate and base. Storage moduli *G′* and loss moduli *G″* were obtained by amplitude sweep with a logarithmic increase in shear strain from 0.01% to 100%. A total of 25 measurement points were recorded for each sample at a constant angular frequency of 10 rad·s^−1^ and at 20 °C. Before each test, a preload of 0.2 N was applied to the gels. All measurements were reproduced three times (n = 3).

#### 4.4.3. Fourier Transform Infra-Red Spectroscopy (FTIR)

FTIR spectroscopy analysis of acellular and cellularized gels after 25 days of culture was performed on a spectrum 400 spectrometer (PerkinElmer, Wellesley, MA, USA). Freeze-dried samples were deposited on an ATR crystal, and transmittance spectra were collected with 32 scans at a resolution of 2 cm^−1^. All the spectra were normalized to type I collagen amide I at 1653 cm^−1^.

### 4.5. Biological Studies

#### 4.5.1. Cell Viability

Cell viability in cell-seeded dense collagen gels was assessed using a Live/Dead^®^ assay (Thermofisher, Waltham, MA, USA) on day 24 post-culture. After rinsing twice with PBS, samples were stained with a 2 µM calcein-AM and 4 µM ethidium homodimer-1 solution and incubated at 37 °C for 30 min. Then, the gels were rinsed three times with PBS to stop the reaction before imaging. Live cells were viewed by green fluorescence and dead cells by red fluorescence using a fluorescent microscope (Axio Imager D.1, Zeiss, Jena, Germany) or a Leica SP5 upright confocal microscope (Leica DMI6000 Upright TCS SP5) under 10× and 20× magnification, respectively. Three image fields were taken for each scaffold and processed with Image J (version 1.53f51).

#### 4.5.2. Metabolic Activity

Cell metabolic activity was assessed using AlamarBlue^®^ Assay (Sigma-Aldrich, St. Louis, MO, USA). Briefly, a 10% FBS-supplemented basal medium (without phenol red), 1% (*v*/*v*) penicillin–streptomycin, and 1% glutamax containing 10% rezazurin was used to replace the culture medium. Samples were incubated at 37 °C for 3 h. Media were collected and replaced with fresh medium to prolong the culture. The reduction percentage of Alamar Blue was calculated from the absorbance values at 570 nm and 600 nm of the collected media following the manufacturer’s instructions.

#### 4.5.3. Histological Studies

After 24 days of culture, the gels were fixed overnight at room temperature in a 4% PFA solution in PBS, followed by a series of graded dehydration with ethanol. Dehydrated constructs were then embedded in paraffin and cut into 7 µm thick sections using a manual microtome (Stiassnie, France). Sections were deparaffinized with Toluene, rehydrated in successive baths of increasing water content, and stained with Masson’s Trichrome (for the presence of cells and collagen), Alizarin Red (Calcium revelation) and Von Kossa (phosphate revelation). Images were acquired using a light microscope (DMLB, Leica).

### 4.6. Statistical Analyses

All quantitative characterizations were performed in triplicate. Data were compared with one-way statistical analysis of variance (ANOVA) tests. *p*-value < 0.05 was considered statistically significant. Data are expressed as mean ± standard deviation (SD).

## Figures and Tables

**Table 1:** Influence of DMEM and NaOH volume and concentration on the macroscopic properties of gels before and after compression using 2 mL of an initial collagen solution 4 mg·mL^−1^ in 20 mM acetic acid.

V_DMEM_ (mL)	[DMEM]	pH	[NaOH] (M)	V_NaOH_ (μL)	Final [DMEM]	Gel before Compression	Gel after Compression
0.5	1X	4.5–5	0.1	350	0.5X	spread and leak	crush
0.5	2.5X	4	0.1	450	0.7X	spread and leak	very thin and fragile
0.5	5X	3.5	0.1	600	0.9X	stable	very thin and stable
0.5	10X	3	0.1	700	1.4X	stable	very thin and stable
1.0	1X	6–7	0.1	0	0.6X	spread and leak	very thin and fragile
1.0	2.5X	3.5	0.1	600	0.8X	stable	very thin and stable
**1.0**	**5X**	**3**	**0.1**	**700**	**1.3X**	**stable**	**thin and stable (H)**
1.0	10X	<3	0.1	>1000	-	-	-
1.5	1X	6–7	0.1	0	0.6X	spread and leak	crush
1.5	2.5X	3.5	0.1	700	0.9X	spread and leak	very thin and fragile
1.5	5X	3	0.1	800	1.6X	spread and leak	crush

**Table 2:** Influence of DMEM and NaOH volume and concentration on the macroscopic properties of gels before and after compression using 2 mL of an initial collagen solution 4 mg·mL^−1^ in 500 mM acetic acid.

V_DMEM_ (mL)	[DMEM]	pH	[NaOH] (M)	V_NaOH_ (μL)	Final [DMEM]	Gel before Compression	Gel after Compression
1.0	1X	3.5–4	5	200	0.3X	spread and leak	very thin and stable
			0.1	1000			
1.0	2.5X	3	5	205	0.7X	stable	thin and stable
			0.1	640			
1.0	5X	2–3	5	210	1.2X	spread and leak	very thin and fragile
			0.1	700			
1.0	10X	2	5	215	2.3X	no gel formed	-
			0.1	450			
1.0	1X	3.5–4	5	200	0.5X	spread and leak	very thin and stable
			1	30			
**1.0**	**2.5X**	**3**	**5**	**205**	**0.8X**	**stable**	**thin and stable (I)**
			**1**	**40**			
1.0	5X	2–3	5	210	1.2X	spread and leak	very thin and stable
			1	60			

## Data Availability

All data and materials are available on request from the corresponding author. The data are not publicly available due to ongoing researches using a part of the data.

## Supplementary Materials

The following supporting information can be downloaded at: https://www.mdpi.com/article/10.3390/gels10020130/s1, Figure S1: additional SEM images; Figure S2: additional Von Kossa staining images; Figure S3: addition Alizarin Red staining images.
