# Supplementary material for: Role of Physico-Chemical and Cellular Conditions on the Bone Repair Potential of Plastically Compressed Collagen Hydrogels
Source: Gels. 2024 Feb 6;10(2):130. doi: 10.3390/gels10020130 (PMC10887598; doi:10.3390/gels10020130)

Supplementary Information

Role of physico-chemical and cellular conditions on the bone repair potential of plastically-compressed collagen hydrogels

Daline Mbitta Akoa, Ludovic Sicard, Christophe Hélary, Coralie Torrens, Brigitte Baroukh,

Anne Poliard, Thibaud Coradin

Figure S1. SEM images of hydrogels prepared in conditions H and I, aged 24 h and compressed.

Figure S2. Von Kossa staining of SHED-cellularized hydrogels after 25 days of culture

Figure S3. Alizarin Red staining of collagen hydrogels by h-DPSCs seeded at two cell densities after 25 days of culture

Figure S1. SEM images of hydrogels prepared in conditions H and I, aged 24 and compressed. Scale bar: 2 μm.

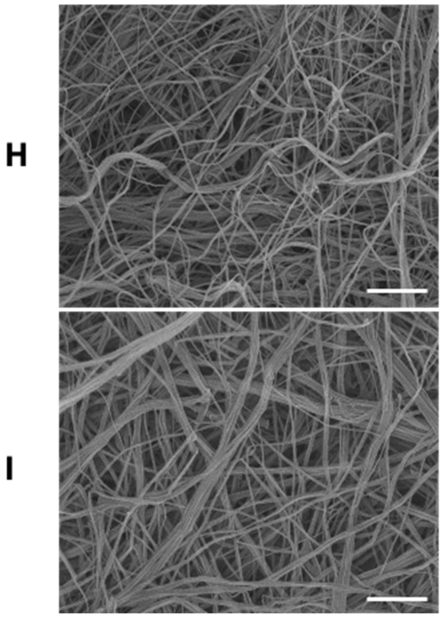

Figure S2. Von Kossa staining of SHED-cellularized hydrogels after 25 days of culture.

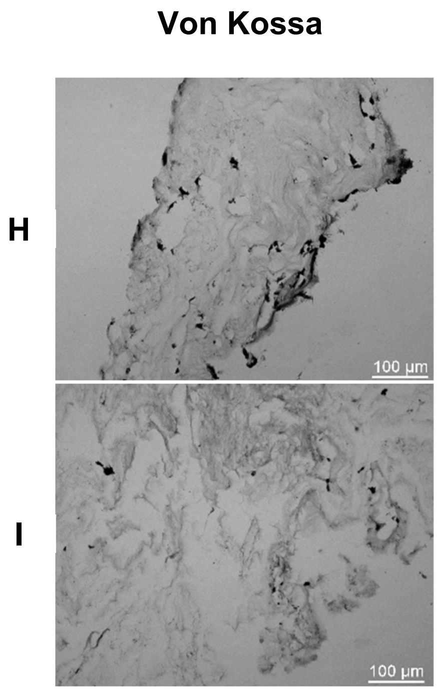

Figure S3. Alizarin Red staining of collagen hydrogels by h-DPSCs seeded at two cell densities after 25 days of culture.

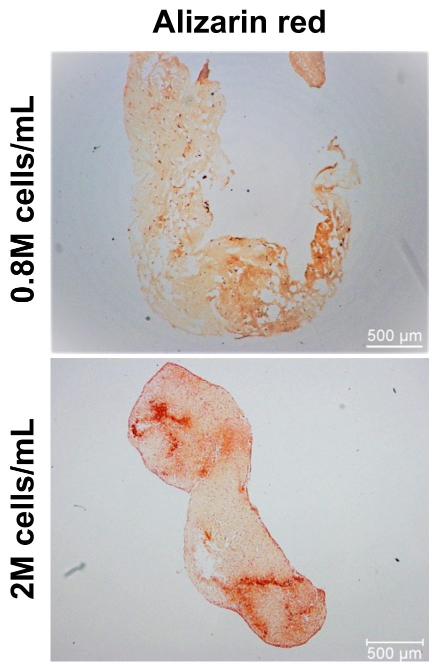

Supplement: Supplementary file 1 [file gels-10-00130-s001.zip › gels-2860832-supplementary.pdf]
